# The impact of severe mental disorders on mother-infant interaction: a systematic review

**DOI:** 10.1007/s00737-025-01561-6

**Published:** 2025-02-06

**Authors:** Berta Vilaseca, Alba Roca-Lecumberri, Cristina García-Gibert, Florencia Forte, Anna Torres-Giménez, Eva Solé, Susana Andrés-Perpiñá, Ana Barajas, Estel Gelabert

**Affiliations:** 1 Department of Clinical and Health Psychology, Universitat Autònoma de Barcelona, Cerdanyola del Vallès, Spain; 2 Unitat de Salut Mental Perinatal CLINIC-BCN, Hospital Clínic, Barcelona, Spain; 3 Fundació de Recerca Clínic Barcelona- Institut d’Investigacions Biomèdiques August Pi i Sunyer (IDIBAPS), Barcelona, Spain; 4 Sexual and Reproductive Health Care Assistance Center, Parc Sanitari Sant Joan de Deu, Cerdanyola del Vallès, Spain; 5 Bipolar and Depressive Disorders Unit, Hospital Clinic of Barcelona, Institute of Neurosciences, IDIBAPS, University of Barcelona, Barcelona, Spain; 6 Biomedical Research Networking Center for Mental Health Network (CIBERSAM – ISCIII), Barcelona, Spain; 7 Faculty of Psychology, University of Barcelona, Barcelona, Spain; 8 Serra Húnter Programme, Generalitat de Catalunya, Barcelona, Spain

**Keywords:** Mother-Infant interaction, Schizophrenia, Bipolar disorder, Social cognition, Postpartum

## Abstract

**Purpose:**

Mother-Infant Interaction (MII) is the first dynamic relationship that focuses on both mother-infant involvement and dyadic coordination and is associated with infant development. The main objective of this review is to summarize the evidence on the quality of MII in mothers with Severe Mental Illness (SMI).

**Method:**

A systematic search for cross sectional, cohort or case control studies has been carried out in PubMed, Web of Science, PsycINFO and Scopus to extract studies addressing the relationship between the quality of MII and SMI.

**Results:**

A total of 15 studies with a sample of 992 women were included. Studies showed worst outcomes for MII in mothers with psychotic disorder and bipolar disorder. The impairments were more pronounced in psychotic disorders.

**Conclusions:**

There is evidence of impaired MII in SMI. Social cognition (SC) is essential for understanding and responding to infant cues, so it could partially explain the associations between SMI and interaction outcomes. The current evidence is limited due to substantial heterogeneity and methodological limitations in the studies. Therefore, such findings should be interpreted with caution.

## Highlights

The co-ocurrence of SMI in the perinatal period presents specific health problems for both mother and child, requiring specialized healthcare. Impairments in MII are more pronounced in psychotic disorders, but there is also an alteration in bipolar disorder. A deeper comprehension of the subject matter would clear the way for the implementation of targeted interventions.

## Introduction

Mother–infant interaction (MII) is the first dynamic and bidirectional relationship that focuses on both mother-infant involvement and dyadic coordination (Beebe et al. 2010). Empirical literature has shown that infants can perceive and be contingent to maternal behavior, effectively synchronizing their interactions with their mothers (Tronick et al. 1977). At the same time, mothers are able to adapt their own behavior in response to the infants’ action (Lewis and Rosenblum 1974). The dyadic systems approach views interaction as a dynamic process, defined by three main factors: maternal contributions (sensitivity and adaptive responses), infant contributions (active organization of behaviors and rhythms), and dyadic coordination (bidirectional adjustments and mutual regulation) (Beebe et al. 2010).

It has been shown that the quality of mother-infant interaction (MII) influences the first years of infant development, both in the cognitive, social, emotional and linguistic domains (Rocha et al. 2020; Soares et al. 2018).

In the clinical population, the quality of MII is a relevant mediator between perinatal mental disorders and infant development (Stein et al. 2014). A considerable amount of research about MII has been focused on the impact of perinatal depression on child development (Soares et al. 2018). In contrast, despite the severity of the symptoms, there has been limited research on the impact of severe mental illness (SMI), including bipolar disorder (BD), schizophrenia (SCZ) spectrum disorders and postpartum psychosis (PP), on early interaction. The International Classification of Diseases 11th Revision (ICD-11) (WHO, 2021) categorizes PP as one of the syndromes associated with pregnancy or the puerperium (i.e., up to about 6 weeks after delivery) that involves mental and behavioral characteristics such as mania, depression, mixed episodes with psychotic features, and unspecified psychosis (Bergink et al. 2016). If the symptoms meet the diagnostic criteria for a specific mental disorder the corresponding diagnosis should be assigned in accordance with the ICD-11 guidelines.

It is well established that individuals with SMI are more likely to experience social vulnerability compared to the general population(Morgan et al. 2018). This vulnerability extends to the perinatal period where mothers suffering from SMI are also at increased risk of relapse (Alcantarilla et al. 2023; Wesseloo et al. 2016). In fact, mothers who committed suicide in the postpartum year were more likely than survivors to have a history of affective disorders, psychotic disorders, and self-harm (Lysell et al. 2018). Similarly, a recent meta-analysis showed that women with puerperal psychosis have a higher incidence of infanticide (Alford et al. 2024). In addition, these mothers also confront a greater susceptibility to experience obstetric complications during pregnancy, delivery, and the early neonatal period, such as preeclampsia, caesarean-section, gestational diabetes or low birth weight (Jablensky et al. 2005; Solé et al. 2020; Tang et al. 2023). Given the additional risks, the pharmacological management presents a significant challenge due to the need to balance maternal mental health and fetal safety, and although certain medications may carry risks, the consequences of not treating mental illness may be more harmful than the potential side effects (Gruszczyńska-Sińczak et al. 2023). As a result, the co-ocurrence of SMI in the perinatal period presents specific health problems for both mother and child, requiring specialized healthcare.

Although much is known about the relationship between the quality of MII and major depressive disorder (MDD) (Slomian et al. 2019), little research has been conducted in the context of SMI. In order to address this gap, we conducted a systematic review to summarize the evidence on the relationship between the quality of MII, as assessed by observational methods, and SMI.

## Materials and methods

This systematic review was performed following recommendations in the PRISMA (Preferred Reporting Items for Systematic Review and Meta-Analysis Protocols) statement(Page et al. 2021). The authors used the PRISMA 2020 update following the checklist item (https://www.prisma-statement.org/prisma-2020-checklist). The protocol for this review was prospectively registered on PROSPERO (CRD42023438889).

### Search strategy

Relevant terms were searched in the databases Scopus, PsycINFO, PubMed and Web of Science databases up to January 2024: (“Postpartum” OR “postpartum period” OR perinatal OR “perinatal period”) AND (Bipolar OR “bipolar disorder” OR “manic-depressive psychos*” OR “bipolar mood disorder*” OR “bipolar affective psychos*” OR “bipolar depression” OR “manic depression” OR “bipolar affective disorder” OR “bipolar spectrum disorder” OR “bipolar illness” OR “psychotic disorder*” OR schizophrenia OR “schizoaffective disorder” OR “Postpartum psychotic disorder*” OR “postpartum psychiatric disorder*”) AND (“Mother infant interaction*” OR “mother child interaction*” OR “mother infant attachment” OR “mother child attachment” OR “mother infant relation*” OR “mother child relation*” OR “maternal bonding” OR “mother infant bonding” OR “maternal sensitivity” OR “dyadic coordination”).

The combination of these terms was searched in the title, abstract and keywords. In addition, the reference list of all eligible studies was screened to identify further additional studies that meet the inclusion criteria. Only articles published in English and Spanish were included.

The search was completed in January 2024; it was re-run just before the final analysis, and studies not identified in previous searches were retrieved for inclusion.

### Eligibility criteria

*Inclusion criter*i.a. were: (i) empirical quantitative studies following cross sectional, cohort or case-control designs which examine MII in postpartum (ii) mothers over 18 years old and up to 16 months postpartum (iii) mothers diagnosed with SMI (diagnosed by a clinical assessment, and interview or using any recognized diagnostic criteria): BD, puerperal psychosis, and psychotic disorders (schizophrenia, schizoaffective disorder, schizophreniform disorder, delusional disorder, brief psychotic disorder, and psychotic disorder not otherwise specified).

*Exclusion criteria* were: (i) case studies, systematic reviews and metanalysis, conference abstracts, letters, commentaries, essays, book chapters, qualitative studies, study protocols, reviews and grey literature (ii) infant participants with severe malformations or serious medical conditions and/or cared for in neonatal intensive care units (iii) studies that were not in English or Spanish were excluded.

### Data extraction

Rayyan (https://www.rayyan.ai/), the online systematic review tool, was used to manage and screen all retrieved papers. Two independent authors (B.V., C.G-G) carried out the two stages of screening based on the title, abstract, and full-text review to identify eligible studies based on inclusion criteria. Any disagreements during study selection were resolved after consultation with a third reviewer (E.G.).

Data were extracted from each study included in the review using a standardized data extraction form in Microsoft Excel. This included information on the following variables: (1) first author, (2) year of publication, (3) country, (4) study design, (5) population size (6) sample description, (7) study measures, (8) instruments used to assess MII, (9) age of infant, (10) interaction domains assessed and (11) results.

### Quality assessment

Two authors (B.V., C.G-C) independently assessed the quality of the studies included, and a third author (E.G.), resolved any disagreements. The quality of the studies was evaluated using Newcastle–Ottawa Scale (NOS) (Wells et al. 2000) for the assessment of the quality of non-randomized studies in systematic reviews and meta-analyses (Table 1).

**Table 1:** Assessment of study quality included in the systematic review by the Newcastle-Ottawa Scale (NOS)

Case-control studies	Case definition (Max.★)	Cases representativeness (Max.★)	Controls selection (Max.★)	Controls definition(Max.★)	Comparability of case- controls based on the design or analysis (Max.★★)	Ascertainment of exposure (Max.★★)	Same method of ascertainment for cases and controls (Max.★)	Non-response rate (Max.★)	Overall quality
Anke et al. 2019	★	★	-	★	★	★	-	-	Poor
Anke et al. 2020	★	★	-	★	★	★	-	-	Poor
Healy et al. 2016	-	-	★	-	★	★	-	-	Poor
Hipwell and Kumar 1996	-	★	-	★	★	-	-	-	Poor
Hipwell et al. 2000	★	★	★	★	★	-	-	-	Poor
Hornstein et al. 2006	★	★	-	★	★	★	-	-	Poor
Logsdon et al. 2015	-	-	-	★	★	-	-	-	Poor
Pawlby et al. 2010	-	★	★	★	★	-	★	-	Poor
Rigby et al. 2016	-	★	-	-	★	★	★	★	Poor
Riordan et al. 1999	-	★	-	★	★	-	★	-	Poor
Snellen et al. 1999	-	★	-	-	-	-	-	★	Poor
Wai Wan et al. 2007	-	★	-	★	★	-	★	-	Poor
Wai Wan et al. 2008	-	★	-	-	★	-	★	-	Poor
Cohort studies	Exposed cohort representativeness (Max.★)	Non-exposed cohort selection (Max.★)	Ascertainment of exposure (Max.★)	Outcome was not present as baseline (Max.★)	Comparability of cohorts based on the design or analysis (Max.★★)	Assessment of outcome (Max.★)	Adequate follow-up period for outcome (Max.★)	Adequacy of follow-up of cohorts (Max.★)	Overall quality
Aran et al. 2021	★	-	★	★	★	★	★	-	Good
Biaggi et al. 2024	★	★	★	★	★	★	★	-	Good

Note. Max, maximum: study can be awarded a maximum of one star for each numbered item within the selection and outcome categories. A maximum of two stars can be given for comparability

## Results

### Study characteristics

**Fig. 1:**
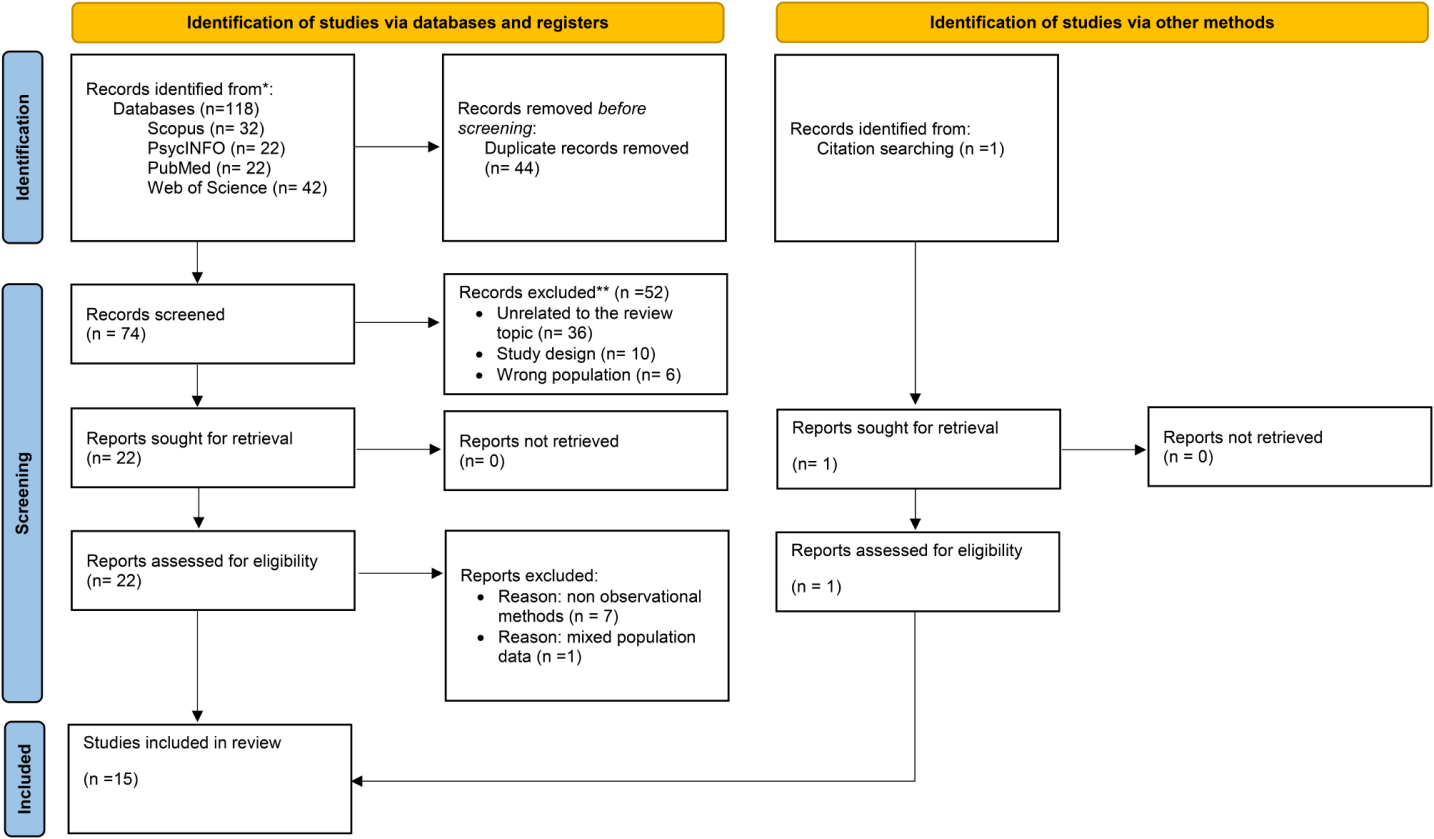
PRISMA flow diagram

Figure 1 summarizes the selection of the articles for review. A total of 118 records were found from all databases, 44 duplicates were removed, leaving 74 records for preliminary screening. Initially, 36 records were excluded based on their titles and abstracts as not relevant to the scope of the review. In addition, 16 articles were excluded for not meeting preliminary eligibility criteria of design type (*n* = 10) and population (*n* = 6), resulting in 22 articles assessed for eligibility. The remaining articles were reviewed based on the inclusion criteria, 7 articles were removed as observational measures were not present in assessing interaction, and 1 was excluded due to the absence of separated data analysis for different mental disorders. We included 1 article identified through citation search. The final number of studies to be included in the review was 15 (Fig. 1). The assessment of study quality is also presented (Table 1).

A brief analysis of the 15 included studies is presented (Table 2), along with an overview of the instruments used to assess MII (Table 3), as well as a summary of the obtained results (Table 4). The results are based on a total sample of 992 women. 9 studies were conducted in UK, 2 in Norway, 2 in Australia, 1 in Germany and 1 in USA. There were 8 prospective observational studies and 7 cross-sectional observational studies.

**Table 2:** Main characteristics of the studies

Author, year	Country	Study design	*N*	Sample Description	Diagnostic criteria	Study measures
(Anke et al. 2019)	Norway	Prospective observational	56	26 BD, 30 HC	Verified and/or by contacting their specialist + semi-structured interview	(a) Sociodemographic; (b) clinical (IDS, YMRS, EPDS, EuropASI, MCMI-III, SCL-25); (c) infant
(Anke et al. 2020)	Norway	Prospective observational	54	7 BD I, 19 BD II, 28 HC	Verified and/or by contacting their specialist + semi-structured interview	(a) Sociodemographic; (b) clinical (IDS, YMRS, EPDS, EuropASI, MCMI-III, SCL-25); (c) infant
(Aran et al. 2021)	Australian	Prospective observational	127	7 BDI, 5 BD II, 60 MDD, 55 HC	SCID-5CV (DSM − 5)	(a) Sociodemographic; (b) clinical (EPDS)
(Biaggi et al. 2024)	UK	Prospective observational	103	43 AR; 60 HC	SCID-I (DSM-IV)	(a) Sociodemographic; (b) clinical (HDRS, YMRS, PANSS); (c) health; (d) obstetric; (e) infant development (Bayley III); (f) GAF; (g) neuropsychological (FSIQ- WAIS-R)
(Healy et al. 2016)	UK	Cross-sectional observational	64	18 SCZ, 24 MDD, 22 HC	Clinical	(a) Sociodemographic; (b) clinical (EPDS, PANSS); (c) affect recognition (PennCNP)
(Hipwell and Kumar 1996)	UK	Prospective observational	78	28 MDD, 35 BD, 15 SCZ	RDC	(a) Sociodemographic; (b) clinical
(Hipwell et al. 2000)	UK	Prospective observational	82	25 severe puerperal mental illness, 16 minor and major depression, 41 HC	RDC, SADS	(a) Sociodemographic; (b) clinical (EPDS)
(Hornstein et al. 2006)	Germany	Cross-sectional observational	35	18 depression, 17 psychotic disorders	SCID-I (DSM-IV), ICD-10	(a) Sociodemographic; (b) clinical (CGI, SOFAS, PANSS, HAMD-21); (c) obstetric; (d) infant; (e) bonding (PBQ)
(Logsdon et al. 2015)	USA	Cross-sectional observational	130	40 BD, 50 MDD, 40 HC	SI-HDRS, clinical	(a) Sociodemographic; (b) clinical
(Pawlby et al. 2010)	UK	Prospective observational	99	23 depression, 15 SCZ, 12 mania, 49 HC	Clinical	(a) Sociodemographic; (b) clinical
(Rigby et al. 2016)	UK	Cross-sectional observational	40	19 depression and anxiety, 10 BD, 7 SCZ, 4 SAD	DSM (SCID)	(a) Sociodemographic; (b) clinical; (c) neuropsychological (NART, WAIS-III verbal fluency); (d) SC (Coat Story, RMET, Frith-Happé Animations)
(Riordan et al. 1999)	UK	Cross-sectional observational	26	8 SCZ, 18 affective disorders	RDC	(a) Sociodemographic; (b) clinical
(Snellen et al. 1999)	Australian	Prospective observational	15	15 psychotic disorders	(*)	(a) Sociodemographic; (b) clinical (PANSS)
(Wai Wan et al. 2007)	UK	Cross-sectional observational	38	13 SCZ spectrum, 14 BD, 11 MDD	ICD-10	(a) Sociodemographic; (b) clinical; (c) infant
(Wai Wan et al. 2008)	UK	Cross-sectional observational	45	23 MDD, 14 SCZ, 8 BD	ICD-10	(a) Sociodemographic; (b) clinical

Note. ^(*)^ Data not provided. AR, Women at Risk of Postpartum because of a diagnosis of bipolar disorder, schizoaffective disorder or previous Psychosis Postpartum; BD, Bipolar Disorder; CGI, Clinical Global Impression; DSM, Diagnostic and Statistical Manual of Mental Disorders; EPDS, Edinburgh Postnatal Depression Scale; EuropASI, European Addiction Severity Index; FSIQ- WAIS-R, full scale IQ- Wechsler Adult Intelligence Scale Revised; GAF, Global Assessment of Functioning; HAMD-21, 21 version Hamilton Depression Scale; HC, Healthy Controls; HDRS, Hamilton Depression Rating Scale; ICD, International Statistical Classification of Diseases and Related Health Problems 10th Revision; IDS, Inventory of Depressive Symptomatology; MCMI-III, Millon’s Clinical Multiaxial Inventory-III; MDD, Major Depressive Disorder; NART, National Adult Reading Test; n.s (not significant); PANSS, Positive and Negative Syndrome Scale; PBQ, Postpartum Bonding Questionnaire; RDC, Research Diagnostic Criteria; RMET, Reading the Mind Eyes Test; SAD, Schizoaffective Disorder; SADS, Schedule for Affective Disorders and Schizophrenia; SCID-5CV DSM 5, Structured Clinical Interview; SCID-I DSM-IV, Structured Clinical Interview; SCZ, Schizophrenia; SI-HDRS, Structured Interview Guide for the Hamilton Scale for Depression; SCL-25, Hopkins Symptom Check List; SCL-90, Symptom Checklist 90; SC, Social Cognition; SOFAS, Social and Occupational Functioning Assessment Scale; YMRS, Young Mania Rating Scale

**Table 3:** Main features of instruments to assess MII

Study(es)	Interaction tool	Evaluation range (months)	Scoring format	Maternal dimension(s)	Infant dimension(s)	Dyadic dimension(s)
Anke et al. 2019 / Anke et al. 2020	Parent–Child Early Relational Assessmentm (PCERA, Clark 1985)	0–60 m	5-point global rating (1 = area of concern, 5 = area of strength)	(a) Maternal positive affective involvement, sensitivity, and responsiveness; (b) Maternal negative affect and behavior	(a) Positive affect, communicative and social skills; (b) Dysregulation and irritability	(a) Dyadic mutuality and reciprocity; (b) Dyadic tension
Aran et al. 2021	Emotional Availability Scales (EAS, Biringen 2008)	0–16 m	9-point global rating scale (3 = worst score, 9 = optimal score)	(a) Sensitivity; (b) Structuring; (c) Non-intrusiveness; (c) Non-hostility	(a) Responsiveness; (b) Involvement	N/A
Biaggi et al. 2024 / Rigby et al. 2016	Child–Adult Relationship Experimental Index (CARE-Index, Crittenden 2007)	0–36 m	3-point global rating (sensitive: 8–14, low sensitive: 5–7, high risk: 0–4)	(a) Sensitivity; (b) Control; (c) Unresponsiveness	(a) Cooperativeness; (b) Difficultness; (c) Compulsiveness; (d) Passivity	(a) Dyadic synchrony
Healy et al. 2016 / Riordan et al. 1999 / Wai Wan et al. 2007	Global Rating Scales (Murray 1988)	0–16 m	5-point global rating (0 = worst score, 5 = optimal score)	(a) Warm; (b) Accepting- rejecting; (c) Responsiveness; (d) Non-demanding; (e) Sensitivity (f) Non-intrusive behavior; (g) Non-Intrusive speech; (h) Non-remote; (i) Non-silent; (j) Happiness; (k) Non-flaccid; (l) Absorbed in infant; (m) Relaxed	(a) Attentiveness; (b) Active communication; (c) Positive vocalizations; (d) Engaged with environment; (e) Liveliness; (f) Happiness; (g) Non-fretful	(a) Smoothness; (b) Fun; (c) Mutually satisfying; (d) Much engagement; (e) Excited engagement
Hipwell and Kumar 1996/ Snellen et al. 1999	Bethlem Mother – Infant Interaction Scale (BMIS, Kumar and Hipwell 1996)	0–12 m	5-point global rating (0 = appropriate, 4 = severe disturbances)	(a) Eye contact; (b) Physical contact; (c)Vocal contact; (d) Mood; (e) General routine; (f) Risk to the baby	(a) Baby´s contribution to interaction	N/A
Hipwell et al. 2000	Play Observation Scheme and Emotion Rating (POSER-Wolke 1986)^(1)^	0–52 m	5-point and 9-point rating scales (*)	(a) Sensitive involvement; (b) Affectionate talk; (c) Maternal control	(a) Expressivity; (b) Task involvement; (c) Level of energy	(a) Quality of interaction
Hornstein et al. 2006	Category system for microanalysis of early MII (Jorg et al. 1994)	(*)	Dichotomous rating (behavior present or absent)	(a) Responsiveness (vocal, facial or motor)	N/A	(a) Appropriate stimulation; (b) Guidance; (c) Control
Logsdon et al. 2015	Ainsworth Maternal Sensitivity Scales (Ainsworth 1969)	Adaptable to a wide range of ages	9-point global rating scale (3 = worst score, 9 = optimal score)	(a) Sensitivity; (b) Cooperativeness (c) Physical and Psychological Availability; (d) Acceptance	N/A	N/A
Logsdon et al. 2015	Dyadic Mutuality Code (DMC)	0–6 m	Dichotomous rating (1 = absent, 2 = present)	(a) Maternal sensitive responsiveness; (b) Maternal pauses	(a) Infant clarity of cues	(a) Mutual attention
Logsdon et al. 2015	Child-Caregiver Mutual Regulation Scale (CCMR, Tronick and Weinberg 1997) ^(2)^	(*)	(*)	(a) Maternal affect; (b) Maternal behavior	(a) Child affect; (b) Child behavior	N/A
Logsdon et al. 2015	Maternal Behavior Q-Sort (MBQS, Moran et al. 2009)	(*)	9-point global rating (higher values indicate optimal scores)	(a) Maternal sensitivity (accessibility, responsiveness, and promptness)	N/A	N/A
Pawlby et al. 2010	MMCS the Mind-mindedness Coding Scheme (MMCS, Meins and Fernyhough 2015)	0–12 m	Coding scheme based on intervals of seconds (frequency).	(a) Responsiveness; (b) Seeks infant’s attention; (c) Interactional pauses; (d) Physic contact	(a) Changes in gaze direction (eyes or head); (b) Changes in gazes to mother	N/A
Snellen et al. 1999	Infant/Caregiver Behavioral Scale (ICBS, Milgrom 1996) ^(3)^	(*)	0-2-point rating scale (0 = occurs zero to 30% of the time, 1 = 30–60%, 2 = > 60%).	(a) Responsiveness; (b) Stimulation; (c) Caregiving; (d) Positive affect; (e) Negative affect; (f) Attention; (g) Soothe	(a) Clarity of cues; (b) Exploration; (c) Smile/excite, (d) Fuss/cry; (e) Attention to other children; (f) Attention to caregiver; (g) Aggression; (h) Alertness	(1) Mutual attention; (2) Reciprocity/synchronicity; (c) Intensity of interaction
Wai Wan et al. 2008	Modified classification system coded (Stanley et al. 2004)	(*)	Coding scheme based on intervals of seconds (frequency)	(a) Affirmative behaviors (affirmation, mirroring, greeting); (b) Negating behaviors (denial, substitution of affect, exaggeration)	(a) Maternal engagement; (b) Protest; (c) Avoidance; (d) Environmental engagement; (e) Dysregulation; (f) Displacement; (h) Gaze ratio	N/A

Note. N/A not applicable. ^(*)^ Data not provided. ^(1)^ Scale description is based on Hipwell et al. (2000) as the manuscript was not published. ^(2)^ Scale description is based on Logsdon et al. (2015) because the complete manual was not available. ^(3)^ Scale description is based on Snellen et al. (1999) as the manuscript was not published

**Table 4:** Main findings of the studies

Author, year	Interaction study measures	Infant age (months)	Results
(Anke et al. 2019)	PCERA	≤ 3 m	(1) Significant group differences on MII with medium to large effect sizes on all subscales, except on (S4). S1 and S6 (*p* = 0.04), S2 (*p* = 0.03), S3 (*p* = 0.01), S5 (*p* < 0.001)
(Anke et al. 2020)	PCERA	3–12 m	(1) Differences with large effect sizes on all subscales except on S6 (0.04), which had a small effect size. S1-S3 (*p* < 0.001), S4 (*p* = 0.001), S5 (*p* < 0.001), S7 and S8 (*p* < 0.001).
(Aran et al. 2021)	EAS	6 m	(1) All of EA dimensions were significantly lower in the BD and MDD groups compared to HC (*p* < 0.001). (2) Women with BD and their infants displayed lower EA than MDD and HC across all maternal and child EA qualities (*p* < 0.001).
(Biaggi et al. 2024)	CARE-Index	2 and 12 m	(1) AR women had significantly less synchronous interactions than HC at 2 (*p* = 0.004) and 12 months (*p* = 0.024). (2) Dyadic synchrony improved significantly from 2 to 12 m in both groups (*p* < 0.001)
(Healy et al. 2016)	GRS	2–16 m	(1) Mothers with SCZ scored significantly less sensitive (*p* = 0.002) less warmth (*p* = 0.01) and less responsive (*p* = 0.016) than HC. (2) Infants of mothers with SCZ showed a trend towards less attentiveness (*p* = 0.032) than infants of MDD mothers.
(Hipwell and Kumar 1996)	BMIS	≤ 6 m	(1) Significant differences were found by maternal diagnostic group (*p* < 0.01), with MDD mothers scoring higher than mothers with BD or psychotic disorder. (2) Significant improvement in appropriateness of maternal behavior, regardless of diagnosis (*p* < 0.001) during admission.
(Hipwell et al. 2000)	POSER	12 m	(1) Mothers with BD were more likely than depressed mothers to vocalize their infant. Infants of BD mothers displayed higher levels of tasks involvement than depressed mothers (*p* < 0.05)
(Hornstein et al. 2006)	Category system for microanalysis of early MII	≤ 8 m	(1) Infants of psychotic mothers showed significantly more avoidance of eye contact than infants of women with MMD (*p* < 0.008).
(Logsdon et al. 2015)	AMSS, MBQS, DMC, CCMR	12 m	(1) BD women had lower scores than HC or MDD mothers; however, none of the differences were significant (n.s).
(Pawlby et al. 2010)	MMCS	≤ 13 m	(1) MDD and BD groups scored significantly higher than the HC in maternal attention seeking (*p* < 0.01) and infant touch (*p* < 0.001). (2) BD group scored significantly higher than the SCZ group in the infant touch (*p* < 0.001).
(Rigby et al. 2016)	Infant CARE-Index	≤ 13 m	(1) Mothers with SCZ were rated the least sensitive compared to mothers with MDD and BD (*p* < 0.000).
(Riordan et al. 1999)	GRS	Around 4 m	(1) SCZ group was more remote, silent, verbally and behaviorally intrusive, self-absorbed, flaccid, insensitive as well as unresponsive and less demanding compared to the affective group (*p* < 0.05). Their infants were more avoidant (*p* < 0.05). The overall interaction was less mutually satisfying, more serious, and with reduced engagement (*p* < 0.05)
(Snellen et al. 1999)	BMIS, ICBS	< 12 m	(1) Significant improvements between admission and discharge for all BMIS variables (*p* < 0.01). (2) In the ICBS, only infant exploration, smile/excitement, alertness, and dyadic mutual attention showed improvement (*p* < 0.05). (3) On admission higher levels of positive symptomatology and mother’s overall psychopathology were correlated with poorer scores (*p* < 0.05). (4) At discharge, the severity of a mother’s negative symptoms correlated with lower scores on MII (*p* < 0.05).
(Wai Wan et al. 2007)	GRS	2–4.5 m	(1) Mothers with SCZ and their infants consistently scored lower than BD and MDD mothers (*). (2) SCZ group compared to affective group were less responsive (*p* < 0.005), less sensitive (*p* < 0.05) and more remote (*p* < 0.005); their infants were more avoidant (*p* < 0.005), less communicative (*p* < 0.05), less engaged (*p* < 0.01) and less lively (*p* < 0.05); and the dyadic interaction was less smooth (*p* < 0.05), less satisfying (*p* < 0.01), with reduced amount of engagement (*p* < 0.005) and less exciting (*p* < 0.05).
(Wai Wan et al. 2008)	Modified classification system coded	2–4.4 m	(1) Mothers with SCZ had a higher rate of non-response due to psychological (*p* = 0.02) and behavioral (*p* = 0.25) withdrawal. (2) Mothers with SCZ were more likely to react negatively to neutral or positive behaviors than mothers with an affective disorder (*p* = 0.08). (3) ‘Abnormal’ maternal responses were rare and only observed in mothers with SCZ (*p* = 0.002).

Note. (*) Data not provided; AMSS, Ainsworth Maternal Sensitivity Scale; BD, Bipolar Disorder; BMIS, Bethlem Mother Infant Observation Scale; CCMR, Caregiver and Child Mutual Regulation System; DMC, Dyadic Mini Code; EA, Emotional Availability; EAS, Emotional Availability Scales; GRS, Global Rating Scales of Mother-Interaction; HC, Healthy Controls; ICBS, Infant/Caregiver Behavioral Scale; Infant CARE-Index, Child-Adult Relationship Experimental Index; MBQS, Maternal Behavior Q-Sort; MDD, Major Depressive Disorder; MII, Mother- Infant Interaction; MMCS, Mind-Mindedness Coding Scheme; n.s (not significant); PCERA, Parent-Child Early Relational Assessment; POSER, Play Observation Scheme and Emotion Rating; SCZ, Schizophrenia

In terms of maternal diagnosis, 8 of the articles reviewed covered BD and psychotic disorders, 4 BD, and 3 psychotic disorders. 7 of the articles had control groups. The assessed infants ranged in age from 0 to 16 months.

All papers included were in English. The results of MII were summarized by diagnostic group.

### Bipolar disorder and mother-infant interaction

Anke et al. (2019, 2020) conducted two prospective longitudinal studies at 3 and 12 months postpartum to compare MII between women with BD and Healthy Controls (HC) using the Parent-Child Early Assessment (PCERA) (Clark 1985). The first study(Anke et al. 2019) found that mothers with BD performed worse than HC at 3 months postpartum on all subscales (*p* = 0.0.4– *p* < 0.001), except on infant dysregulation and irritability (S4). The differences between BD and HC were most significant for dyadic mutuality and reciprocity (S5) (*p* < 0.001). Concurrent symptom load and quality of interaction were not significantly associated. These results are consistent with those obtained at 12 months postpartum(Anke et al. 2020). Significant differences remained for all subscales (S1-S5: *p* = 0.001– *p* < 0.001; S6: *p* = 0.04).

### Bipolar disorder versus major depressive disorder and mother-infant interaction

Three studies (Hipwell et al. 2000; Logsdon et al. 2015; Aran et al. 2021) compared MII between women diagnosed with BD and MDD. The first study (Hipwell et al. 2000) evaluated the MII at 12 months in two case groups and a control group: an inpatient case group (severe mental disorders admitted to a specialized mental health unit), an outpatient case group (non-psychotic unipolar depression who continued to live at home); and a HC group (mentally healthy mothers). The MII was coded using the Play Observation Scheme and Emotion Rating (POSER)(Wolke 1986) [Unpublished manuscript], and principal component analyses were performed on the maternal behaviors (sensitive involvement, affectionate talk, maternal control), child behaviors (expressivity, task involvement, level of energy) and joint behaviors (quality of interaction) items. The data used in this review resulted from the division of two diagnostic groups (MDD and BD) of the inpatient group. BD mothers were more likely than MDD mothers to talk affectionately to their infants (*p* < 0.05). Infants of BD mothers displayed higher levels of task involvement than depressed mothers (*p* < 0.05). These differences exist when comparing diagnoses, but not when comparing with HC.

A second study (Logsdon et al. 2015), compared MII between women with BD or MDD and controls. Four observational instruments were used to assess MII: Ainsworth Maternal Sensitivity Scale (AMSS)(Ainsworth 1969), Maternal Behavior Q-Sort (MBQS) (Moran et al. 2009) [Unpublished doctoral dissertation], The Dyadic Mini Code (DMC) (Censullo et al. 1987), and the Child-Caregiver Mutual Regulation Scale (CCMR)(Logsdon et al. 2015; E. Z. Tronick and Weinberg 1997). The authors found that BD mothers had lower scores than MDD or HC, but none of the differences were statistically significant.

More recently, Aran et al. (2021), conducted a prospective study in which the MII was assessed using the Emotional Availability Scales (EAS) (Biringen 2008). All dimensions of emotional availability were found to be significantly lower in the BD and MDD groups compared to HC (*p* < 0.001). Across the entire range of maternal qualities, maternal sensitivity was the one that differed the most between mothers with disorder (BD, MDD) and HC. Furthermore, women with BD showed more impairment than those of MDD and HC dyads, in all maternal (sensitivity, structuring, non-intrusiveness, non-hostility) and infant (responsiveness, involvement) domains.

### Psychotic disorders and mother-infant interaction

Three studies explored the relationship between diagnosis of psychotic disorder and MII difficulties.

Snellen et al. (1999) evaluated the MII in women diagnosed with SCZ, schizophreniform disorder and brief psychotic disorder using The Infant/Caregiver Behavioral Scale (ICBS) (Milgrom 1996) [Unpublished manuscript] and The Bethlem Mother-Infant Interaction Scale (BMIS) (Hipwell and Kumar 1996). The ICBS includes 18 behavioral measures, consisting of seven maternal measures, eight infant measures and three joint mother-infant measures. The BMIS measures seven variables: (a) eye contact, (b) physical contact, (c) vocal contact, (d) mother’s mood, (e) general routine, (f) risk to baby, (g) baby’s condition. In line with the aim of the study, the first four measures that fall under the “dialogue score (a-d)” along baby’s contribution (g) have been used. The authors found a significant improvement between admission and discharge for all BMIS variables except for Baby Care (*p* < 0.01). In contrast, of the ICBS, only infant exploration, smile/excitement, alertness, and dyadic mutual attention showed significant improvement (*p* < 0.05). The authors also found that higher levels of maternal positive symptomatology and overall psychopathology on admission were correlated with poorer MII scores (*p* < 0.05). At discharge, the severity of a mother’s negative symptoms, especially the anergic aspects, correlated with lower scores of MII (*p* < 0.05).

A second study (Hornstein et al. 2006) compared the MII between women admitted to a Mother Baby Unit (MBU) with a diagnosis of MDD and those with SCZ spectrum disorders. The authors used the categorical system for micro-analysis of the early MII (Jorg et al. 1994) to assess maternal responsiveness (vocal, facial or motor) and interactive behavior (stimulation, guidance and control). This study found that babies of psychotic mothers showed significantly more avoidance of eye contact than babies of women with MMD (*p* = 0.008).

More recently, Healy et al. (2016) investigated the impact of affect recognition on the quality of MII in women diagnosed with SCZ compared to women with MDD and HC. They used Global Ratings Scales (GRS) (Murray 1988) to evaluate MII, which assessed two main domains: maternal behavior and infant behavior. Maternal behavior was rated on 7 dimensions: sensitivity, warmth, acceptance, responsiveness, non-demanding, non-intrusive, non-remote. Infant behavior was rated on 3 dimensions: attentive, lively and happy. The authors concluded that mothers with SCZ scored significantly lower in sensitivity (*p* = 0.001), warmth (*p* = 0.014) and responsiveness (*p* = 0.016) compared to HC. Infants of SCZ mothers showed a trend towards less attentiveness (*p* = 0.032) compared to the infants of MDD mothers. This study also assessed affect recognition with the Emotions Battery of the University of Pennsylvania Computerized Neuropsychological Test (PennCNP)(Kohler et al. 2003). The authors found that impairments in emotion recognition tasks partially predicted the number of bizarre/unusual communication (*p* = 0.007). Bizarre comments included statements with content that was either odd or unusual; statements that were clearly inappropriate for the child’s developmental stage (e.g., emphasizing the chest as the most important body part) and making unusual or frightening noises (e.g., growling). Additionally, a diagnosis of SCZ significantly enhanced the ability to predict the occurrence of such statements (*p* = 0.005).

### Psychotic disorders versus affective disorders (MDD and BD) and mother-infant interaction

This section includes four studies that analyzed data on BD alongside other affective diagnoses, therefore it was not possible to make comparisons between mothers with BD and SCZ.

The first of them(Riordan et al. 1999) compared the interaction in women with SCZ and affective disorders (MDD, BD, minor depressive disorder) at the time the mothers were about to be discharged from hospital. The assessment of MII was conducted at the time of the highest level of functioning, including for women with chronic illnesses. For women admitted with an acute illness, it followed a period of clinical recovery, typically after successful week of home leave. They used GRS (Murray 1988) to evaluate MII, which assessed three main domains: maternal behavior, infant behavior and interaction. Both dimensions of maternal and infant behavior were described in the study by Hornstein et al. (2006). Interaction was rated on 5 dimensions: smooth, fun, mutually satisfying, much engagement and exciting engagement. Mothers with SCZ displayed more significant difficulties in interacting with their children than those with an affective disorder. They were significantly less responsive, less sensitive as well as more intrusive, silent, self-absorbed and flaccid (*p* ≤ 0.05). Their children were significantly more avoidant, and overall interaction was less fun, less satisfying and less engaged (*p* ≤ 0.05).

Wan et al. (2007, 2008) replicated these results in two longitudinal studies that used different interaction assessment tools. The first study (Wan et al. 2007) found that mothers with SCZ and their infants achieved the least optimal interaction scores than those with BD and MDD. Subsequently, the interaction scores of the GRS were compared between SCZ and affective disorders (MDD and BD). Compared to the affective disorders group, women with SCZ were less responsive (*p* < 0.005), less sensitive (*p* < 0.05) and more remote (*p* < 0.005); their infants were more avoidant (*p* < 0.005), less communicative (*p* < 0.05), less engaged (*p* < 0.01) and less lively (*p* < 0.05); and the dyadic interaction was less smooth (*p* < 0.05), less satisfying (*p* < 0.01), with reduced amount of engagement (*p* < 0.005) and less exciting (*p* < 0.05). According to the authors, a diagnosis of SCZ was a stronger predictor of poor MII than illness severity or poor social stability.

In this instance, Wan et al. (2008) employed a different and more specific instrument to assess MII. This instrument consisted of a modified rating system, previously described by Stanley et al. 2004 (Stanley et al. 2004) and originally developed to assess MII in a sample of mothers with MDD. The results showed that mothers with SCZ had a higher rate of non-response due to psychological (*p* = 0.02) and behavioral (*p* = 0.25) withdrawal compared to the affective group. Additionally, mothers with SCZ were more likely to react negatively to neutral or positive behaviors than mothers with an affective disorder (*p* = 0.08) and abnormal maternal responses were rare and only observed in mothers with SCZ (*p* = 0.002).

Recently, a prospective longitudinal study (Biaggi et al. 2024) was conducted to follow a group of women who were at-risk-of-postpartum psychosis (AR) due to a diagnosis of BD, schizoaffective disorder (SAD), SCZ, and postpartum psychosis, as well as HC. In particular, the AR women had the following diagnoses: 33 (76.7%) bipolar disorder, 6 (14%) schizoaffective disorder, and 4 (9.3%) previous PP. The authors reported that 41.9% of all women experienced a relapse (AR-unwell), while 58.1% remained symptom-free (AR-well) within 4 weeks of delivery. The study was conducted from 25 weeks’ gestation to 12 months postpartum and the quality of MII was evaluated with the Child-Adult Relationship Experimental Index (Infant CARE-Index) (Crittenden 2007). [Unpublished manuscript] at 8 weeks and 12 months. The Infant CARE-Index assesses three adult scales (Sensitivity, Control and Unresponsiveness) and four infant scales (Cooperation, Compulsivity, Difficultness, Passivity) as well as Dyadic Synchrony. The study authors chose to select Maternal Control, Infant Compulsiveness, Infant Difficultness and Dyadic Synchrony. AR women had significantly less synchronous interactions than HC at 8 weeks (*p* = 0.024) and 12 months postpartum (*p* = 0.037). Additionally, dyadic synchrony improved significantly in both groups from 8 weeks to 12 months (*p* < 0.001). There were no significant differences in synchronous interactions between the AR well and AR unwell groups.

### Psychotic disorder versus bipolar disorder and mother-infant interaction

In 1996, Hipwell & Kumar examined the quality of MII among women admitted to a MBU. The study used the BMIS to assess MII at three different time points: one week after admission, two weeks after admission, and the week of discharge. The rating of subscale baby’s contribution to the quality of interaction was not included. The total sample of women was classified according three diagnostic groups: psychotic disorders, BD and MDD. Significant differences were found by maternal diagnostic group (*p* < 0.01), with MDD mothers scoring higher than mothers with bipolar or psychotic disorder. Over the course of admission, the mothers showed a significant improvement in appropriateness of maternal behavior, regardless of diagnosis (*p* < 0.001). At discharge, the majority of BMIS scores in women with MDD and BD were rated in the normal range (86% vs. 77% respectively). In contrast only 33% of SCZ women fell into the same category.

Such findings were replicated by Rigby et al. (2016) which explored the relationship between the theory of mind (ToM) and MII in women with SMI (depression and anxiety, BD, SCZ, SAD). The MII was assessed through Infant CARE-Index and the authors concluded that mothers with SCZ were rated the least sensitive compared to mothers with MDD and BD (*p* < 0.000). One ToM task, the Frith–Happé Animations, predicted maternal sensitivity across all diagnoses. There was also an effect of diagnosis, with lower sensitivity observed in women with SCZ. ToM impairments did not fully explain the effect of diagnosis on sensitivity. Mothers of girls were rated as being more sensitive than mothers of boys.

One of the weaknesses of previous studies is the lack of a control group. In 2010, Pawlby et al. conducted a study to investigate the impact of SMI according to three diagnostic groups: SCZ, depression (depressive mood disorders with or without psychosis) and mania (mood disorders where mania was the predominant feature, with or without psychosis) on MII in comparison to HC. The study found that both the depression and mania groups were more intrusive scoring significantly higher than the HC on maternal attention seeking (*p* < 0.01) and infant touch (*p* < 0.001). Additionally, the mania group scored significantly higher than the SCZ group in the infant touch (*p* < 0.001).

## Discussion

The results of the systematic review suggest that impairment in MII is associated with both BD and SCZ spectrum disorders. The results suggest that mothers with psychotic disorders show greatest impairment in MII. In all the studies, mothers with affective disorders show better interactions outcomes than mothers with psychosis. Specifically, mothers with BD have better interaction with their babies than mothers with psychotic disorders, but worse than mothers with MDD.

The review highlights that woman with BD scored significantly lower compared to HC across all domains of the MII at 3 months (Anke et al. 2020), 6 months(Aran et al. 2021), and 12 months postpartum(Anke et al. 2019). Additionally, less affectionate and less involvement tasks were observed(Hipwell et al. 2000). There was also a trend towards less maternal sensitivity and infant reciprocity at 12 months, although the differences did not reach significance(Logsdon et al. 2015).

In relation to psychotic disorders, several studies reported that mothers with SCZ exhibit less sensitivity, responsiveness, and warmth compared to mothers with affective disorders (Riordan et al. 1999; Wan et al. 2007, 2008) and HC(Healy et al. 2016) with one exception (Pawlby et al. 2010). This is true not only for mothers with SCZ but also for those with diagnoses related to the SCZ spectrum (Snellen et al. 1999).

Furthermore, mothers without mental disorders performed consistently better across all dimensions of maternal interaction, except in one study (Pawlby et al. 2010) where no significant differences were observed when compared to clinical groups (MDD, BD and SCZ). According to the authors, one reason for these results may be that the Mind-mindedness Coding Scheme (MMCS) (Meins and Fernyhough 2015) [Unpublished manuscript] was developed to assess general population. No association between symptom-load and interaction quality was found in BD (Anke et al. 2019, 2020; Biaggi et al. 2024) or psychotic disorders (Healy et al. 2016; Riordan et al. 1999; Wan et al. 2007, 2008). This suggests that other factors independent of affective or psychotic symptomatology may influence MII.

In an attempt to explain these results some have referred to social cognition (SC) as a potential mechanism that may partially explain the associations between SMI and interaction outcomes (Anke et al. 2019, 2020; Healy et al. 2016; Rigby et al. 2016; Wan et al. 2007, 2008). SC is a multidimensional construct that includes a set of cognitive processes related to detect, process and interpret the intentions and behaviors of others that support adaptive social behavior (Harvey and Penn 2010). In 2006 the National Institute of Mental Health (NIMH) classified five main domains within this construct: theory of mind (ToM), attributional bias, emotional processing, social perception and social knowledge(Green et al. 2008). Most studies examined the relationship between SC and both SCZ and BD reported significant deficits on the specific domains of ToM(Gillissie et al. 2022; Yeh et al. 2021) and emotional processing (DeTore et al. 2018; Martins et al. 2019; Varo et al. 2017, 2022). In addition, particularly in the case of BD, there is evidence of deficits both in the active (Samamé 2013) and remission phases (de Siqueira et al., 2020; Samamé et al. 2012).

Indeed, it has been reported that SC is more likely to be impaired in SCZ than in BD (Bora and Pantelis 2016) and, in turn, patients with BD have worse outcomes on tasks assessing SC than patients with MDD (van Neerven et al. 2021). This might be related to the attainment of poorer interactional scores in SCZ compared to BD, and better outcomes in MDD compared to BD. In this line, there is evidence that interventions focused on ToM within the context of SMI can improve MII (Kenny et al. 2013; Schacht et al. 2017).

The ability of mothers to engage in sensitive and responsive interactions implies emotion recognition and mentalizing skills, particularly when interacting with infants, who have limited language repertoires and subtle emotional cues. There is evidence that perinatal period (pregnancy and postpartum) hormone land neurobiological changes may increase maternal sensitivity and responsiveness to emotional cues (Barba-Müller et al. 2019). Thus, given that SC has been shown to predict social and functional outcomes in SCZ(Pinkham 2014; Silberstein and Harvey 2019) and BD(Varo et al. 2017; Vlad et al. 2018) future research should investigate whether it may be a strong predictor of the quality of MII.

It is therefore important to note that deficits in SC are present in SMI and have a significant impact on MII. Future research should prioritize understanding how neurobiological changes during the puerperium can enhance SC, as well as developing early and targeted interventions to address SC deficits. Such interventions could have the potential to improve maternal and infant outcomes, as well as long-term outcomes in SCZ and BD.

### Strengths and limitations

To the best of our knowledge this is the first systematic review to gather evidence on MII in the context of SMI based on observational instruments. We conducted a search strategy, an independent review and assessed the quality of the studies.

The study is subject to some limitations. First, the methodological heterogeneity across the studies in terms of the observational instruments used to assess MII, age of the child at assessment and diagnostic groupings. Second, the number of selected studies was limited. Third, studies samples have a very wide range of symptom severity. Studies reporting psychopathological stability do not use the same criteria. This is even more pronounced in the case of SCZ. Thus, many studies include bipolar and depressive disorders under the umbrella of affective disorders which reduces the conclusions that can be drawn about MII in the context of maternal BD. Lastly, several studies were judged to be at high risk of bias, with concerns about case definition, selection of controls, ascertainment of exposure and non-response date.

## Conclusions

There is evidence of impaired MII in mothers with SMI. The impairments are more pronounced in psychotic disorders, but there is also an alteration in BD. There may be other factors, apart from symptomatology, that could explain the results. A deeper comprehension of the subject matter would clear the way for the implementation of targeted interventions designed to improve maternal and child well-being.

The current evidence is limited due to substantial heterogeneity and methodological limitations in the studies. Therefore, such findings should be interpreted with caution.
